# The Cedar Project: Relationship between child apprehension and attempted suicide among young Indigenous mothers impacted by substance use in two Canadian cities

**DOI:** 10.1371/journal.pone.0252993

**Published:** 2021-06-10

**Authors:** Lisa Ritland, Victoria Thomas, Kate Jongbloed, David S. Zamar, Mary P. Teegee, Wenecwtsin-Kukpi Christian, Chris G. Richardson, Martin Guhn, Martin T. Schechter, Patricia M. Spittal, for the Cedar Project Partnership

**Affiliations:** 1 Human Early Learning Partnership, School of Population and Public Health, University of British Columbia, Vancouver, BC, Canada; 2 BC Children’s Hospital Research Institute, Vancouver, BC, Canada; 3 Wuikinuxv Nation; 4 School of Population and Public Health, University of British Columbia, Vancouver, BC, Canada; 5 Takla Lake First Nation, Carrier Sekani Family Services, Prince George, BC, Canada; 6 Centre for Health Evaluation and Outcome Sciences, Providence Health Care, Vancouver, BC, Canada; 7 Splatsin te Secwepemc; Simon Fraser University, CANADA

## Abstract

Indigenous leaders are gravely concerned over disproportionate representation of Indigenous children in Canada’s child welfare systems. Forced separation from children is deeply traumatizing for mothers and detrimental to the wellbeing of Indigenous families, communities and Nations. This study examined relationships between child apprehension and suicide attempt within a cohort of young Indigenous women impacted by substance use. We utilized data collected every 6 months (2008–2016) by the Cedar Project, an Indigenous-governed cohort study involving young Indigenous people who use drugs in British Columbia, Canada. Recent child apprehension was defined as having a child apprehended by the Ministry of Child and Family Development since last visit. Recurrent event Cox proportional hazards models estimated the independent effect of child apprehension on maternal suicide attempt. Among 293 participants, 78 (27%) reported 136 child apprehensions; incidence of first apprehension was 6.64 (95%CI: 5.25–8.29) per 100 person-years. Forty-seven (16%) participants reported 75 suicide attempts with an incidence of 4.00 (95%CI: 2.94–5.33) per 100 person-years. Participants who reported recent child apprehension (HR: 1.88, 95%CI: 1.00–3.55), had a parent attend residential school (HR: 4.12, 95%CI: 1.63–10.46), experienced recent sexual assault (HR: 4.04, 95%CI: 2.04–7.99), violence (HR: 2.54, 95%CI: 1.52–4.27) or overdose (HR: 4.97, 95%CI: 2.96–8.35) were more likely to attempt suicide. Participants who had a traditional language spoken in the home growing up were half as likely to attempt suicide (HR: 0.49, 95%CI: 0.23–1.01). Results suggest that child welfare systems in Canada perpetuate historical and intergenerational trauma among young Indigenous mothers. Indigenous self-determination over child welfare and culturally safe services are urgently needed to end cycles of child apprehension and support the wellbeing of families, communities and Nations.

## Introduction

Indigenous knowledge keepers share that foundations of health and wellness of Indigenous peoples extend beyond the individual to include all relations, including family, community and Nation, as well as kinship connections to land, water, and all living things [1–3]. In this perspective of wholistic relationality, children are considered gifts from the Creator and raising them is a sacred responsibility [4–6]. Children play a critical role in cultural continuity of Indigenous communities; they have the right to live in their traditional territory, as well as maintain their traditional language and cultural identity [7]. Prior to colonization, traditional laws existed to keep children safe. Indigenous systems of care were in place, for example, to arrange fostering and adoption with community or extended family [8].

In contrast, the wellbeing of Indigenous children, families and Nations are profoundly harmed when children are uprooted from their communities and traditional territories. European settler colonialism in Canada has sought to displace Indigenous peoples from their land and extinguish their languages and culture. Several eras of settler-colonial laws and policies enacted by Canadian governments have amounted to over 140 years of legislated apprehension of Indigenous children. Between 1874–1996, government and church authorities forcefully removed more than 150,000 Indigenous children from their families and communities into the Indian Residential School system [9]. The primary intent of residential schools was to assimilate and sever Indigenous children from their communities, culture, and identity [9, 10]. From the 1960s-1980s, estimates suggest that 20,000 Indigenous children were taken into the child welfare system [11]. Widely described as the “Sixties Scoop,” this systematic, nation-wide policy placed Indigenous children in foster care or adoption arrangements outside of their communities [11]. Intergenerational Residential School and Sixties Scoop survivors face significant risk of having their own children apprehended, compounding losses across generations. Indigenous children comprised almost half (48%) of children in foster care in 2011, but represented only 7% of the general child population [12]. Indigenous leaders have characterized overrepresentation of Indigenous children in contemporary child welfare systems as the “Millennial Scoop” [13]. In 2015, the Truth and Reconciliation Commission of Canada (TRC) heard testimonies from survivors and their families of epidemic physical and sexual abuse, neglect and death in residential schools, as well as the perpetuation of injustice in contemporary child welfare systems. The TRC has mandated the federal, provincial, territorial, and Indigenous governments to reduce the number of Indigenous children in care [14].

Overrepresentation of Indigenous children in child welfare systems has severe consequences for health and wellbeing, including social determinants of health [15]. Indigenous peoples carry disproportionate burden of poor health and disease in Canada, produced by social, political, economic and cultural inequalities related to historic and present-day colonization [16]. Child apprehension experiences are both a consequence of, and contributor to, negative social determinants of health across the life course and across generations. Children and youth in foster care are more likely to become street-involved and have poorer health outcomes later in life compared to their peers [17, 18]. The vast majority of Indigenous families are pushed into child welfare systems as a result of substance use, poverty, and inadequate housing [19, 20]. Indigenous mothers in Canada are also disproportionately targeted by “birth alerts”, which allow child welfare systems to apprehend newborns immediately after birth without parental knowledge or consent [21]. In addition, Indigenous mothers face significant barriers to regaining custody of their children [22]. In 2016, the Canadian Human Rights Tribunal (CHRT) ruled that child welfare systems in Canada discriminate against Indigenous children and families living on reserve [23]. The CHRT ordered Canada to immediately reform child welfare systems by providing equal funding for Indigenous children on reserves, as well as culturally safe early supports and prevention services to address impacts of historical and intergenerational traumas.

Alarming rates of suicide among Indigenous young people in North America are entwined with the legacy of colonization and ongoing systemic violence [24–26]. In the province of British Columbia (BC), the suicide rate among status First Nations youth aged 15–24 was 2.77 per 10,000 population between 2011–2015. This is significantly higher than rates (0.79 per 10,000 population) among other young people living in BC [27]. A review of unexpected deaths between 2010–2015 among First Nations youth and young adults (age 15–24 years) in BC demonstrated that 32% (n = 30) died by suicide [28]. Furthermore, a quarter (27%, n = 8) of those who took their own lives were parents of young children [28]. Maria Yellow Horse Brave Heart first described historical trauma among the Lakota people in the United States as “cumulative emotional and psychological wounding” caused by colonization, including displacement from traditional land, removal of children in boarding schools, and legal suppression of cultural and spiritual practices [29]. Unresolved grief from historical and intergenerational trauma contribute to concerning sequelae documented in empirical literature, including substance use, Post-Traumatic Stress Disorder (PTSD), depression and anxiety, sexual abuse, family violence, anger, low self-esteem and suicide [30–35].

Emerging evidence also suggests that the trauma of separation from children through child welfare systems has a life-changing impact on mother’s health and wellness, including among those affected by substance use. After having a child apprehended by the state, women affected by substance use report deteriorating mental health, symptoms of PTSD and psychological distress, and increased drugs and alcohol use to cope with loss and grief [36–41]. Since 2016, BC has struggled with a public health emergency from pervasive levels of fentanyl in the local drug supply, which has resulted in rising opioid-related overdose deaths. A recent study found marginalized women in Vancouver, Canada who had their children apprehended experienced greater odds of unintended, non-fatal drug overdose and these odds were even greater for Indigenous women [42]. In the context of economic precarity, impacts on social determinants of health may also include loss of child-related income supplements with consequences for housing and food security. Symptoms of trauma and substance use are often cyclical and compounded by separation from children taken into foster care [37, 38].

Despite adversities, Indigenous communities and young people have long resisted removal of their children and demonstrated resilience through upholding cultural systems and connections to territories, kin, and identity. Resilience is a strengths-based and culturally safe construct for understanding how Indigenous peoples overcome historical and intergenerational trauma [3, 43, 44]. Indigenous perspectives on resilience are diverse and informed by distinct cultures, languages, and spirituality together with relationships, shared histories and environments [45]. Indigenous scholars have conceptualized community resilience as the process of healing from historical trauma and cultural loss from colonization, through recognizing trauma impacts and promoting cultural identity [46]. Cultural loss is a theorized mechanism resulting in Indigenous young people rejecting life, while research demonstrates cultural continuity may protect against suicide risk [47]. Others have called to re-conceptualize resilience as Indigenous political resistance to ongoing adversity, which provides a more relevant framework to recognize community strengths and promote wellbeing [48]. In addition, it has been said that motherhood is powerful medicine, affirmed by research indicating that motherhood can represent a turning point and is motivation to seek treatment for trauma and substance use [38, 39].

Previous research from the Cedar Project indicates that having a parent attend residential school, child welfare involvement, and childhood sexual abuse are associated with long-term negative health outcomes within a cohort of Indigenous young people who have used drugs [49, 50]. However, few studies have considered the harmful impact of having a child apprehended on the wellbeing of Indigenous mothers navigating substance use and multiple traumas. To our knowledge, no previous epidemiological studies have longitudinally explored associations between child apprehension, cultural strengths, and attempted suicide among young Indigenous mothers. This study aimed to (a) describe incidence of state-based child apprehension and (b) examine the relationship with suicide attempt among young Indigenous women who have used drugs in Vancouver and Prince George, BC, Canada. In addition, a range of protective and risk factors for suicide attempt were examined including historical and lifetime trauma, cultural connectedness, and drug- and sexual-related vulnerabilities.

## Methods

### The Cedar Project

The Cedar Project (Cedar) is governed by the Cedar Project Partnership (the Partnership), a group of Indigenous Elders, leaders, experts, and scholars. The Partnership assures ethical standards are met, informs research design and paradigm, identifies analyses and interprets results, controls communication and media strategies, and develops recommendations. This study followed the principles of Ownership, Control, Access, and Possession (OCAP®) and the Tri-Council Policy Statement: Ethical Conduct for Research Involving Humans with emphasis on Chapter 9, related to research involving Indigenous peoples. The University of British Columbia Human Ethics Board approved the study (certificate number H17-02201) on February 23, 2018.

At enrolment, all participants met with an Indigenous Study Coordinator (the Coordinator) who described the study, confirmed eligibility, and requested informed consented after explaining potential benefits and harms associated with participation. After the age of 14, minors may consent for themselves regarding their own medical care, therefore consent is not sought from parents or guardians. All participants receive a copy of the consent form and staff verbally review the full consent form with participants. The consenting process may take from 10 to 60 minutes depending on the participant and the number of questions for staff about the study. It is vital for all staff to review the consent form with participants outlining participant rights throughout the research process and explaining what will occur at every stage. Nothing should come as a surprise to participants throughout the duration of their involvement with Cedar.

Cedar is an Indigenous-governed prospective cohort study involving 793 young Indigenous people who have used drugs in Vancouver and Prince George, BC, Canada [51]. Baseline data was collected between 2003–2005 in Vancouver and Prince George, and then again in 2011 when enrollment was re-opened. Eligible participants completed interviewer-administered questionnaires measuring sociodemographic characteristics, injection and non-injection drug use, sexual risk, service utilization, and mental and social issues. The Coordinator made clear that participants had the option to be interviewed by an Indigenous person if preferred or by a trusted confidant to protect confidentiality in small communities. Venous blood samples were drawn and tested for HIV and hepatitis C infections. Participants also completed pre/post-test counselling with a trained nurse who could refer them to HIV and hepatitis C care. Interviewers and nurses carried out follow-up questionnaires, blood testing, and pre/post-test counselling every six months. While participants were encouraged to return for their test results, this was not a requirement. Participants received an honorarium after each study visit.

### Sample

Eligibility criteria stipulated that participants must self-identify as Indigenous, be between ages 14–30, and have smoked or injected illicit drugs including methamphetamine, crack, heroin, cocaine or other drugs in the month prior to enrolment. Saliva Screens (Oral-screen, Avatar Onsite Diagnostics) were administered to confirm drug use. Indigenous identity was defined as descending from First Nations Peoples of North America and included Métis, Aboriginal, First Nations, Inuit and Status and non-Status Indians. Participants were recruited through referral by health workers, community outreach, and word of mouth. The present analysis was restricted to female participants who returned for follow-up visits from January 2008 to October 2016, since longitudinal information on child apprehension was not collected prior to 2008. Among 405 female participants, 37% were excluded because they did not return for at least two follow-ups in the study period. There were no significant differences in mean age (p = 0.472), parental residential school attendance (p = 0.336), injection drug use (p = 0.436) or number of suicide attempts (p = 0.105) among participants who were excluded. In addition, there was no significant difference in the number of participants who reported ever being pregnant (p = 0.748) or having a child apprehended (p = 0.313) among those who did not return for follow-up visits.

### Measures

Variables were selected based on previous methodological or empirical considerations. Time-varying variables were collected during follow-up visits every six months (‘recent’) unless otherwise specified.

#### Recent child apprehension

The primary predictor variable was recently having a child apprehended by the child welfare system. We defined recent child apprehension as having a child apprehended by the Ministry of Child and Family Development (MCFD) since last study visit. Cedar nurses asked participants during a pre-test counselling interview at each study visit, “Since your last visit, have any of your children been apprehended by the Ministry of Child and Family Development?” Response options were yes, no, unsure, and refused. Informal custody loss (children living apart from the mother without MCFD intervention) was not examined in this study.

#### Suicide attempt

The primary outcome variable was recent suicide attempt. Cedar nurses asked participants during a pre-test counselling interview during each study visit, “Have you attempted suicide in the last 6 months?” Response options were yes, no, unsure, and refused. Cedar staff receive suicide intervention training to determine immediacy of suicidal thoughts and/or behaviour. Participants who were suicidal agreed to a verbal contract not to hurt themselves and were referred to additional services for psychiatric care.

#### Cultural connectedness

Culture variables were a secondary variable of interest conceptualized by two Indigenous Elders who are traditional knowledge keepers and members of the Cedar Project Partnership [49]. We defined cultural connectedness in this analysis as having a traditional language spoken in the home growing up. Interviewers asked participants at each study visit, “When you were growing up, how often was your traditional language spoken in your house?” Response options were always, often, rarely, never, unsure, and refused. We created a time-invariant variable to preserve the number of cases. First, participants who ever answered “always” or “often” to the question were coded as always/often. Second, the remaining participants who ever answered “never” or rarely” to the question were coded as rarely/never.

#### Intergenerational and historical trauma

Intergenerational and historical trauma were secondary variables of interest defined as having at least one parent who attended residential school or the participant ever being taken from biological parents themselves. Interviewers asked participants at baseline, “Do you know if your biological parents attended residential school?” and “Were you ever taken from your biological parents?”

#### Other variables

Time-invariant variables included: education (less than high school/high school or higher), sexual identity (LGBTQ2S/heterosexual), interview location (Vancouver/Prince George), age when first taken from biological parents (years), ever forced to have sex (yes/no), age of first sexual abuse (years), and age of first non-injection drug use (years). Childhood sexual abuse was defined as any type of sexual activity that was forced or coerced (including, molestation, rape and sexual assault). Time-invariant indicators of mental health included ever thought about suicide (yes/no), ever attempted suicide (yes/no), and ever self-harmed (yes/no).

Time-varying variables included: age (years), relationship status (single/in a relationship), initiation of injection drug use (yes/no), age of first injection drug use (years), frequency of drug use (less than daily/daily or more), binge drinking (yes/no), overdose (yes/no), homelessness (yes/no), jail/incarceration (yes/no), recent sexual assault (yes/no), recent violence (yes/no), sex work (yes/no), and receiving alcohol or drug treatment (yes/no). Binge drinking was defined as drinking alcohol more than usual. Overdose was defined to include both intentional and unintentional overdose. Homelessness was defined as having spent at least three nights on the street or not having anywhere to go for three days in a row or longer. Jail/incarceration was defined as having been in detention, jail, or prison overnight or longer. Recent sexual assault was defined as being touched where you are not supposed to be without your consent or forced to have sex against your will. Violence was defined being attacked, assaulted, or experiencing any kind of violence without your consent. Sex work (yes/no) was considered an indicator of a sexual vulnerability. Sex work was defined as receiving money, goods, drugs, shelter or anything else in exchange for sex.

### Analysis

Incidence of first event of child apprehension and suicide attempt were calculated using person-time methods. Since Cedar questionnaires evaluate suicide attempt within the past six months, the exact date of each suicide attempt is unknown and was estimated as the mid-point of the previous 6 months. Extended Cox’s proportional hazards regression model for recurrent events proposed by Anderson and Gill [52] estimated the independent effect of child apprehension on parental suicide attempt. Robust variance estimation was used to adjust the variance of all model coefficients to account for within-subject correlation, using a sandwich method similar to that introduced by Lin and Wei [53]. All analysis was carried out using R version 4.0.2 [54], and survival package [55]. Unadjusted and adjusted odds ratios with standard errors and 95% confidence intervals are provided. Models were adjusted for potential confounders, including age and location. Variables found significant in univariate analysis were included in a stepwise multivariate Cox model with entry criteria p<0.10. The multivariate final model was obtained via backward and forward variable selection based on the Wald test as the likelihood and score tests assume independence of the observations and do not account for within-participant correlation. Variables with p<0.20 were retained during the forward selection process. Variables with p>0.10 were removed during the backward selection process. Random bootstrap sampling with 100 replications was performed to validate covariate selection in the multivariate analysis [56]. Proportional hazards were confirmed by examining plots of the scaled Schoenfeld residuals against transformed time for each covariate [57].

## Results

Among 293 eligible participants, 212 (74%) reported ever being pregnant and 206 (70%) were mothers at the first study visit (Table 1). Among mothers, 107 (52%) reported ever having a child apprehended by the Ministry of Child and Family Development. Only 23 (11%) had all of their children living with them. At first study visit, 37 (18%) of mothers were caring for at least one of their children, 54 (26%) had at least one child in foster care, 52 (25%) had at least one child living with the mother’s family, 30 (15%) had at least one child who was adopted, 21 (10%) had at least on child living with the father, and 28 (14%) had children with other living arrangements.

**Table 1:** Child apprehension and child living arrangements among female participants (n = 293) at first study visit, 2008–2016.

Variable	n (%)
**Ever been pregnant**	
Yes	212 [72%]
No	59 [20%]
Unsure/Refused/Missing	22 [8%]
**Ever had a child apprehended by the Ministry of Child & Family Development**	
Yes	107 [37%]
No, but has children	99 [34%]
No children	51 [17%]
Unsure/Refused/Missing	36 [12%]
**Where children are living at first study visit^1^**	
With mother	37 [18%]
With father	21 [10%]
With mother’s family	52 [25%]
With father’s family	35 [17%]
Foster care	54 [26%]
Adopted	30 [15%]
Other	28 [14%]

^1^Categories are not mutually exclusive (e.g. participants can be included in more than one category if they have more than one child).

At first study visit, median age of participants was 27 years old (IQR: 24–30). Overall, 163 (56%) resided in Prince George and 129 (44%) resided in Vancouver (Table 2). There were 137 (47%) participants whose parent(s) had attended residential school and 209 (72%) had been involved with the child welfare system as children. Median age that participants were first separated from their biological parents was 5 years old (IQR: 2–8). A total of 193 participants (66%) reported that they had experienced sexual abuse and median age of first sexual abuse was 6.5 years old (IQR: 5–10). Half (51%) had ever thought about suicide and just less than half (44%) had attempted suicide prior to the study period. Many participants also experienced homelessness (66%), incarceration (50%), and sex work involvement (68%). At baseline, 243 (88%) of participants had used non-injection drugs recently. Median age of first non-injection drug use was 16 years old (IQR: 13–18) and 168 (58%) had ever injected drugs. Additionally, 98 (34%) had experienced an overdose before the study period. Almost three-quarters (73%) had ever received alcohol or drug treatment. Half (52%) reported having a traditional language spoken at home growing up.

**Table 2:** Comparison of baseline characteristics among female participants who reported suicide attempt (n = 61) and those who did not (n = 226) during the study period, 2008–2016.

Variable	Units	Suicide Attempt	No Suicide Attempt	Total
		n (%)	n (%)	n (%)
Demographic				
Interview location	Vancouver	16 [26%]	110 [49%]	129 [44%]
	Prince George	45 [74%]	116 [51%]	163 [56%]
Age	(Median, IQR)	27 [23, 29]	27 [24, 30]	27 [24, 30]
Education	High school or more	9 [16%]	36 [16%]	45 [16%]
	Less than high school	49 [84%]	186 [84%]	239 [84%]
Sexual orientation	Heterosexual	46 [75%]	195 [86%]	145 [84%]
	LGBTQ2S	15 [25%]	32 [14%]	48 [16%]
Relationship status	In a relationship	33 [54%]	116 [52%]	150 [52%]
	Single	28 [46%]	106 [48%]	138 [48%]
Trauma				
Either parent attended residential school	No	12 [20%]	72 [32%]	85 [29%]
	Yes	37 [60%]	99 [44%]	137 [47%]
	Unsure	12 [20%]	54 [24%]	69 [24%]
Ever removed from biological parents	No	13 [34%]	68 [30%]	83 [28%]
	Yes	48 [79%]	158 [70%]	209 [72%]
Age removed from biological parents	(Median, IQR)	5 [1, 7]	5 [2, 9]	5 [2, 8]
Ever forced to have sex	No	14 [23%]	81 [36%]	96 [34%]
	Yes	46 [77%]	143 [64%]	193 [66%]
Age of first sexual abuse	(Median, IQR)	7 [5, 11.5]	6 [4, 10]	6.5 [5, 10]
Ever thought about suicide	No	17 [30%]	106 [50%]	125 [46%]
	Yes	40 [70%]	105 [50%]	148 [54%]
Ever attempted suicide	No	21 [37%]	134 [64]	162 [56%]
	Yes	36 [63%]	77 [36]	129 [44%]
Ever self-harmed	No	23 [40%]	127 [60%]	154 [57%]
	Yes	34 [60%]	83 [40%]	118 [43%]
Ever slept on the street for >3 nights	No	18 [30%]	77 [34%]	98 [34%]
	Yes	42 [70%]	148 [66%]	192 [66%]
Ever in prison overnight	No	32 [53%]	109 [48%]	145 [50%]
	Yes	29 [48%]	117 [52%]	147 [50%]
Substance use				
Age at first non-injection drug use	(Median, IQR)	14 [13, 17]	16 [13, 18]	16 [13, 18]
Ever injected drugs	No	29 [48%]	93 [41%]	124 [42%]
	Yes	32 [52%]	133 [59%]	168 [58%]
Age at first injection drug use	(Median, IQR)	17 [15, 20]	17 [15, 21]	17 [15, 20]
Ever overdosed	No	30 [49%]	156 [70%]	186 [65%]
	Yes	31 [51%]	66 [30%]	98 [35%]
Sexual vulnerability				
Ever involved in sex work	No	17 [28%]	73 [32%]	92 [32%]
	Yes	44 [72%]	152 [67%]	199 [68%]
Protective factors				
Traditional language spoken in house growing up	Never/rarely	23 [40%]	93 [45%]	118 [44%]
	Always/often	34 [60%]	114 [55%]	151 [56%]
Ever had alcohol or drug treatment	No	12 [20%]	61 [27%]	76 [26%]
	Yes	47 [80%]	164 [73%]	213 [74%]

Over the 8-year study period (1174 person-years), 78 (27%) participants who returned for at least two follow-ups reported a combined total of 136 child apprehensions. Person years were used to take into account the number of years each participant was enrolled in the study and calculate the incidence of child apprehensions and suicide attempts during the study period. The incidence rate was 6.64 (95%CI: 5.25, 8.29) child apprehensions per 100 person-years. In total, 47 participants (16%) who returned for at least two follow-ups reported a combined total of 75 suicide attempts. The corresponding incidence rate was 4.00 (95% CI: 2.94–5.33) suicide attempts per 100 person-years. Table 3 reports factors associated with time-to-suicide attempt in bivariate cox regression analyses. Variables in the bivariate analysis were adjusted for age and location.

**Table 3:** Proportional hazards models of predictors of attempted suicide among female participants, 2008–2016.

Variable	Units	Unadjusted			Adjusted		
		CI.95					
		P-value					
		HR	CI.95	P-value	HR	CI.95	P-value
**Demographics**							
Age		0.90	[0.84;0.97]	0.003	0.92	[0.86;0.99]	0.019
Interview location	Vancouver	Ref			Ref		
	Prince George	2.75	[1.25;6.02]	0.012	2.32	[1.02;5.27]	0.044
Relationship status	In a relationship	Ref			Ref		
	Single	1.09	[0.63;1.87]	0.763	1.13	[0.66;1.91]	0.660
Sexual orientation	Heterosexual	Ref			Ref		
	LGBTQ2S	2.28	[1.27;4.11]	0.006	2.31	[1.29;4.15]	0.005
Education	High school or more	Ref			Ref		
	Less than high school	0.76	[0.31;1.85]	0.540	0.50	[0.22;1.18]	0.114
**Trauma**							
Either parent attended residential school	No	Ref			Ref		
	Unsure	0.99	[0.40;2.46]	0.979	1.06	[0.42;2.69]	0.903
	Yes	1.96	[0.89;4.31]	0.093	1.99	[0.92;4.29]	0.080
Ever removed from biological parents	No	Ref			Ref		
	Yes	1.93	[0.89;4.16]	0.095	1.69	[0.75;3.78]	0.204
Age removed from biological	No	Ref			Ref		
parents above cohort median (<5)	Yes	0.78	[0.38;1.58]	0.486	0.97	[0.48;1.98]	0.943
Ever sexually abused		1.45	[1.01;2.07]	0.042	1.56	[1.10;2.23]	0.014
Age of first sexual abuse above	No	Ref			Ref		
cohort median (<7)	Yes	1.01	[0.47;2.16]	0.988	1.29	[0.59;2.80]	0.525
Child apprehended since last visit	No	Ref			Ref		
	Yes	1.99	[1.05;3.77]	0.036	1.89	[1.01;3.53]	0.045
Experienced violence (recent)	No	Ref			Ref		
	Yes	3.64	[2.22;5.98]	<0.001	3.53	[2.18;5.69]	<0.001
Sexually assaulted (recent)	No	Ref			Ref		
	Yes	5.26	[2.83;9.75]	<0.001	5.61	[3.11;10.12]	<0.001
Homelessness (recent)	No	Ref			Ref		
	Yes	2.03	[1.19;3.47]	0.010	2.01	[1.16;3.47]	0.012
Incarceration (recent)	No	Ref			Ref		
	Yes	1.68	[0.93;3.04]	0.088	1.55	[0.85;2.83]	0.151
**Substance use**							
Age at first non-injection drug use	No	Ref			Ref		
above cohort median (<16)	Yes	0.68	[0.36;1.29]	0.241	0.93	[0.46;1.89]	0.847
Ever injected drugs	No	Ref			Ref		
	Yes	3.57	[1.19;10.76]	0.024	3.15	[1.03;9.63]	0.044
Injection drug use (recent)	No	Ref			Ref		
	Yes	1.38	[0.79;2.43]	0.260	1.64	[0.93;2.88]	0.086
Age at first injection drug use	No	Ref			Ref		
above cohort median (<17)	Yes	0.76	[0.31;1.82]	0.532	0.63	[0.21;1.89]	0.415
Daily non-injection crack (recent)	No	Ref			Ref		
	Yes	0.79	[0.42;1.49]	0.472	1.01	[0.54;1.87]	0.976
Daily non-injection crystal meth	No	Ref			Ref		
(recent)	Yes	2.24	[1.09;4.59]	0.028	1.86	[0.91;3.78]	0.088
Daily injection opiates (recent)	No	Ref			Ref		
	Yes	0.90	[0.49;1.63]	0.720	0.94	[0.52;1.73]	0.851
Daily injection cocaine (recent)	No	Ref			Ref		
	Yes	1.23	[0.37;4.11]	0.737	2.86	[0.77;10.63]	0.117
Daily injection crystal meth (recent)	No	Ref			Ref		
	Yes	2.28	[1.02;5.10]	0.046	2.17	[0.98;4.78]	0.055
Daily injection heroin (recent)	No	Ref			Ref		
	Yes	0.88	[0.46;1.70]	0.706	0.95	[0.49;1.86]	0.885
Overdose (recent)	No	Ref			Ref		
	Yes	6.66	[4.05;10.95]	<0.001	5.53	[3.30;9.27]	<0.001
Binge alcohol (recent)	No	Ref			Ref		
	Yes	1.60	[0.84;3.02]	0.150	1.68	[0.89;3.16]	0.110
**Sexual vulnerability**							
Involved in sex work (recent)	No	Ref			Ref		
	Yes	2.10	[1.24;3.55]	0.006	2.13	[1.26;3.59]	0.004
**Protective factors**							
Traditional language spoken in	Never/Rarely	Ref			Ref		
house growing up	Always/Often	0.67	[0.36;1.26]	0.217	0.61	[0.33;1.13]	0.117
Alcohol or drug treatment (recent)	No	Ref			Ref		
	Yes	1.12	[0.66;1.90]	0.679	1.26	[0.75;2.11]	0.380

In multivariable cox regression analysis (Table 4), participants who reported recent child apprehension were almost twice as likely to attempt suicide (HR: 1.88, 95% CI: 1.00–3.55), accounting for potential confounders. Participants whose parent(s) attended residential school were over four times more likely to attempt suicide (HR: 4.12, 95%CI: 1.63–10.46). Furthermore, participants who experienced recent sexual assault (HR: 4.04, 95%CI: 2.04–7.99), violence (HR: 2.54, 95%CI: 1.52–4.27), and overdose (HR: 4.97, 95%CI: 2.96–8.35) were more likely to attempt suicide. Participants who were older (HR: 0.89, 95%CI: 0.83–0.95) and who had a traditional language spoken at home growing up (HR: 0.49, 95%CI: 0.23–1.01) were less likely to attempt suicide.

**Table 4:** Multivariable stepwise analysis of variables associated with suicide attempt during the study period among female participants, 2008–2016.

Variable	Units	HR	CI.95	p-value
Age		0.89	[0.83;0.95]	< 0.001
Location	Vancouver	Ref		
	Prince George	2.27	[0.85;6.03]	0.100
Overdose (recent)	No	Ref		
	Yes	4.97	[2.96;8.35]	< 0.001
Sexual assault (recent)	No	Ref		
	Yes	4.04	[2.04;7.99]	< 0.001
Experienced violence (recent)	No	Ref		
	Yes	2.54	[1.52;4.27]	< 0.001
Child apprehended since last visit	No	Ref		
	Yes	1.88	[1.00;3.55]	0.050
Either parent attended residential school	No	Ref		
	Unsure	1.40	[0.55;3.52]	0.478
	Yes	4.12	[1.63;10.46]	0.003
Traditional language spoken in house growing up	Never/Rarely	Ref		
	Always/Often	0.49	[0.23;1.01]	0.052

## Discussion

This study demonstrates that young Indigenous mothers who use drugs in BC are having their children apprehended by the Ministry of Child and Family Development at an alarming rate. We observed an incidence rate of 6.64 child apprehensions per 100 person-years. It is important to recognize that Cedar participants are intergenerational survivors of the residential school system, and survivors of the Sixties Scoop. Findings from this study demonstrate that, now as parents, they are also profoundly impacted by the Millennial Scoop [58, 59]. Apprehensions reported in this study represent a continuation of nearly 150 years of federal and provincial legislation and policy targeting Indigenous families and kinship structures. Ongoing apprehensions of Indigenous children has consequences for health and wellbeing, as previous evidence from the Cedar has demonstrated that child welfare involvement is associated with long-term negative health outcomes [49, 50].

### Child apprehension

Young Indigenous women who recently had a child apprehended were almost twice as likely to attempt suicide, even after controlling for demographics, past and recent traumas, substance use, and sexual vulnerabilities. Our findings contribute to an emerging literature examining the harms of having children removed on the health and wellbeing of young Indigenous mothers entrenched in substance use and complex trauma. This study provides evidence affirming what is common sense—that separating mothers from their children adversely impacts the health and wellbeing of mothers. These findings are highly relevant in light of the nation-wide overrepresentation of Indigenous children in child welfare systems. Substantial evidence demonstrates that concerning proportions of apprehensions are linked to economic hardship and/or funding structures that incentivize removals rather than prevention [19, 60, 61]. Self-determined culturally safe prevention resources for families, including trauma-and-violence-informed approaches to support healing from lifetime and intergenerational traumas, are urgently required [62, 63].

Indigenous leaders have called for jurisdictional control over child welfare services as an inherent right, to protect their children and families [64]. The Splatsin people of the Secwepemc Nation in BC have been the first and only Indigenous government to assert jurisdiction over child welfare legislation through a band by-law in 1980. The Splatsin people were alarmed by the high proportion of their children being apprehended and placed in homes outside of their community. The legislation recognized the exclusive Splatsin jurisdiction over Indigenous child custody proceedings according to Splatsin laws, traditions and customs [65]. Since 2016, the federal government has been slow to implement the reforms outlined in the CHRT ruling and has received multiple non-compliance orders [66]. Recently, the federal government passed Bill C-92, which aims to recognize Indigenous peoples’ jurisdiction over child welfare. This is a promising step; however, it is essential that it is accompanied by adequate funding directly to Indigenous communities (rather than provincial child welfare bodies). Indigenous jurisdictional control over child welfare will support revitalization of child protection processes and address social determinants of child health and wellbeing for Indigenous families [63].

### Suicide risk

Young Indigenous women in this study reported a rate of 4 suicide attempts per 100 person-years. This figure corroborates decades-old research concluding Indigenous young people attempt suicide to end feelings of grief and loss, which arises from ongoing colonialism, disadvantage, and an unjust relationship with the state [25, 26]. Our findings indicate that cycles of trauma contribute to suicide risk among Indigenous women who have used drugs, particularly the intergenerational impacts of residential schools, sexual assault, and violence. Participants who had a parent attend residential school were 4.12 times more likely to attempt suicide than those who did not. As well, participants who recently experienced sexual assault were 4.04 times more likely to attempt suicide, while those who recently experienced physical violence were 2.54 times more likely. Previous Cedar research indicates that young Indigenous women who have used drugs are more likely to attempt suicide [67] and are overrepresented among suicide deaths [68] compared to their male counterparts. These findings speak to the intersection of gender in the complex role of historical and lifetime trauma in suicide risk among Indigenous young people. In this study, young Indigenous women who reported overdosing in the past six months were 4.97 times more likely to attempt suicide. Previous Cedar research identifies overdose as the method of as many as 35% of suicide attempts [67]. Young Indigenous women in this study may therefore be overdosing as a method to take their own lives. Dismantling institutional structures and policies that harm Indigenous women entrenched in substance use are therefore integral to suicide prevention efforts for this population. Indigenous women must also be actively engaged in developing trauma-informed, violence-informed and culturally safe services.

To our knowledge, this is the first empirical study to affirm that traditional language enculturation may protect against suicidal behaviour among young Indigenous women affected by substance use. Participants who reported a traditional language was spoken in their home growing up were half as likely to attempt suicide during the study period. Cedar research has previously used epidemiological methods to affirm what Indigenous Elders have known since time immemorial: that traditional ways of knowing, language and culture foster resilience among young Indigenous people [49]. Indigenous leaders and scholars have long argued that cultural identity is the foundation of mental wellness and suicide prevention for Indigenous young peoples [1, 69–71]. Walters, Simoni and Evans-Campbell (2002) incorporate cultural resilience in an ‘Indigenist’ stress-coping model to understand the impact of historical and contemporary discrimination in relation to Indigenous women’s health and mental health outcomes. The effect of life stressors (e.g. historical and lifetime trauma) on Indigenous women’s health are moderated by protective factors such as family and community, spirituality, traditional healing practices and Indigenous identity [71]. Empirical studies have also shown that First Nations communities in BC with higher indicators of traditional culture and language knowledge (fluent speaking abilities) have lower rates of youth suicide [47, 72]. Many Indigenous communities throughout BC and Canada are using cultural and language revitalization to respond to suicide in their communities [73]. It is critical that cultural wellness programs are adequately resourced and available for young people who may be living away from home communities including in urban areas such as Vancouver and Prince George.

### Limitations

This study was subject to some limitations typically encountered in cohort studies. First, random sampling was not feasible due to the hard-to-reach nature of this population, which may limit the generalizability of results. Second, findings are based on self-reported data, therefore recall bias, and social desirability bias are possible. Third, while our study adjusted for potential confounders, unmeasured confounding may explain some of the observed associations. Despite these limitations, findings from other studies exploring suicide risk and protective factors among Indigenous young people suggest results are likely generalizable to other contexts in BC and Canada.

## Conclusion

Overrepresentation of Indigenous children in child welfare systems in Canada is a public health and human rights issue. The rate of child apprehensions among Indigenous mothers who have used drugs in this study is alarming and must be viewed as a continuation of cycles of colonial apprehensions that began with the residential schools and endure into the present. This study emphasizes that young Indigenous mothers who have used drugs continue to be affected by historical and present-day injustices and face barriers to maintaining custody of their children. Child apprehension is harmful to the health of these mothers, who are more likely to attempt taking their own lives after recent separation from their children. Child apprehension must therefore be understood as a negative social determinant of Indigenous peoples’ health. Despite these challenges, Indigenous spirituality, language, tradition and culture have endured and serve as the foundation of resilience and resistance among young Indigenous women who have used drugs. Stopping cycles of apprehension and respecting Indigenous self-determination over child welfare is critical to the future health of Indigenous children, families and communities.
